# Evaluation of the Antidiarrheal Activity of Hydromethanol Crude Extracts of *Ruta chalepensis* and *Vernonia amygdalina* in Mice

**DOI:** 10.1155/2020/8318713

**Published:** 2020-04-13

**Authors:** Amsalu Degu, Belayneh Kefale, Diriba Alemayehu, Gobezie Temesgen Tegegne

**Affiliations:** ^1^School of Pharmacy and Health Sciences, United States International University-Africa, Nairobi, Kenya; ^2^Department of Pharmacy, College of Health Sciences, Debre Tabor University, Debre Tabor, Ethiopia; ^3^Department of Pharmacy, College of Medicine and Health Sciences, Ambo University, Ambo, Ethiopia; ^4^Department of Pharmacology and Clinical Pharmacy, School of Pharmacy, College of Health Sciences, Addis Ababa University, Addis Ababa, Ethiopia

## Abstract

**Background:**

The herbal medicine practitioners in Ethiopia have used a wide range of medicinal plants as antidiarrheal agents. Among these, *Ruta chalepensis* and *Vernonia amygdalina* were claimed to have antidiarrheal activity in Ethiopian folklore medicine. Hence, the aim of the present study was to evaluate the antidiarrheal activity of the crude extracts of *Ruta chalepensis* and *Vernonia amygdalina* in mice.

**Methods:**

The crude extracts were obtained by cold maceration with 80% methanol, and its antidiarrheal activities were evaluated using a castor oil-induced diarrheal model. The test groups were treated with 100, 200, and 400 mg/kg body weight (bw) of the crude extract of each plant, while the positive controls and negative controls were given loperamide (3 mg/kg.bw) and 2% Tween 80 (10 ml/kg.bw), respectively.

**Results:**

In the castor oil-induced model, the crude extract of *Ruta chalepensis* (200 and 400 mg/kg.bw) significantly prolonged the onset of diarrhea in mice. Besides, it also showed a significant reduction in the frequency of stooling and weight of feces. Contrastingly, the crude extract of *Vernonia amygdalina* had a significant effect in delaying the onset of time of diarrhea and reduction of the frequency of stool and the weight of feces only at the maximum tested dose (400 mg/kg.bw).

**Conclusion:**

The present study demonstrated that the crude leaves extract of *Ruta chalepensis* (200 and 400 mg/kg.bw) and *Vernonia amygdalina* (400 mg/kg.bw) possessed significant antidiarrheal activity in the castor oil-induced diarrheal model.

## 1. Background

Despite the advancement of medicine, diarrheal diseases are still the second leading cause of death among children under the age of five. [1] Globally, herbal medicines account for 80% of the healthcare needs of the majority of developing countries. [2] Even though the drug manufacturing industry has developed a large number of drugs, indigenous phytotherapy is still practiced in several rural areas, using treatments handed down from generation to generation. [3] In Ethiopia, about 80% of populations relied on traditional medicine due to the cultural acceptability, relatively low cost of traditional medicine, and limited access to modern health facilities. [4].

Despite advances of science in the understanding of the causes, treatment, and prevention of diarrheal diseases, significant number of children's death occur every year. [5, 6] Even if sufficient drugs are available for treating diarrhea, the majority of the existing drugs suffer from adverse effects like the induction of bronchospasm, vomiting (racecadotril), intestinal obstruction, constipation (loperamide) [7], and dependency (diphenoxylate). [8] Because of this, there is a necessity of strengthening research into medicinal plants to investigate alternative drugs from natural products. [9].

Furthermore, the use of medicinal plants for the treatment of diarrhea in folk medicine is a routine practice in many countries of the world. However, in most of the cases, these practices were handed down from generation to generation empirically without knowing the plausible mechanisms, safety, and efficacy of herbal treatments. [10] Among these plants, the leaves extract of *Vernonia amygdalina and Ruta chalepensis* has a claimed folklore use as an antidiarrheal agent. [11, 12] Besides, the previous study by Olayemi et al. showed that the ethanolic stem bark extract of *Vernonia amygdalina* had significant antidiarrheal activity. [13] Besides, the methanolic aerial parts extract of *Ruta chalepensis* had significant antisecretory activity on cholera toxin-induced intestinal secretion in a rat jejunal loops model. [14] Despite this traditional claim and previous studies, it was not fully investigated scientifically to validate its therapeutic effect. Hence, the present study aimed to scientifically validate its importance to corroborate the traditional claim in Ethiopia.

## 2. Methods

### 2.1. Drugs and Chemicals

Castor oil (Amman Pharmaceutical Industries), loperamide (Daehwa Pharmaceuticals), distilled water, Tween 80 (Atlas Chemical Industries Inc.), and methanol (Fisher Scientific, UK) were used in the study.

### 2.2. Plant Material

Fresh leaves of *Ruta chalepensis* and *Vernonia amygdalina* were collected from the Gondar and Gedo districts in October 2018. A taxonomist (Professor Sebsebe Demissew) authenticated the plant, and a voucher specimen (number AD002 and AD002) was deposited at the National Herbarium of Addis Ababa University for future reference. The collected leaves were cleaned using tap water to remove dust particles. Later on, the leaves were dried under shade at room temperature and then rumpled to a coarse powder using a mortar and pestle.

### 2.3. Experimental Animals

Swiss albino mice of either sex with a weight of 20–30 g and age of 6–8 weeks were used for the experiment. The animals were sourced from the animal house of the Department of Pharmacy, Ambo University. The animals were kept in the medium plastic cages (measures 28 cm length, 17 cm width, and 15.5 cm height) at room temperature with a 12h light-dark cycle, relative humidity of 30%, and free access to pellet food and water *ad libitum*. Before the experiment, the animals were acclimatized to the laboratory condition for one week. [14] All the experiments for this study were done following the guideline for the care and use of laboratory animals [15].

### 2.4. Preparation of the Crude Extract

Three hundred grams of powdered plant material of each plant was macerated in an Erlenmeyer flask with 80% methanol for a period of 72 h with occasional shaking using an orbital shaker. It was then filtered two times with Whatman filter paper (NO.1). To increase the percentage yield of the extract, the residue was remacerated two times for a total of 6 days. Then, methanol was removed from the filtrate using a rotary evaporator. Finally, the filtrate was placed in an oven to remove water. After drying and removal of methanol, the percentage yield of the semisolid crude leaves extracts of *Ruta chalepensis* and *Vernonia amygdalina* was found to be 26.7% and 23.3%, respectively.

### 2.5. Grouping and Dosing

The mice were randomly allocated into five groups of six animals per group. The first group was assigned as a negative control and administered 2% Tween 80 at a dose of 10 ml/kg.bw. The second group was assigned as a positive control and administered with standard drug loperamide at a dose of 3 mg/kg.bw. The rest of the groups were treated with 100, 200, and 400 mg/kg.bw of the crude extract. Effective dose selection was based on an oral acute toxicity study of the crude extract as well as pilot experiments. Since there were no observed signs and symptoms of toxicity at 2000 mg/kg.bw, 1/20^th^, 1/10^th^, and 1/5^th^ of 2000 mg/kg.bw doses were used for the actual experiment. The extract was reconstituted with 2% of Tween 80 during the day of the experiment and administered orally.

## 3. Determination of Antidiarrheal Activity

### 3.1. Castor Oil-Induced Diarrhea

In order to determine the antidiarrheal activity of the medicinal plants, the method described by Umer et al. [15] was employed for this study. Swiss albino mice of either sex were deprived of food with free access to water for 18 h. Then, the mice were randomly allocated and treated with their respective doses. One hour later, all the mice were given 0.5 ml of castor oil orally with oral gavage. Then, the mice were separately placed in cages with the floor lined with transparent paper and changed every hour. The mice were carefully followed for 4 h to measure the time of onset of diarrhea, frequency of defecation, and weight of feces excreted by the animals. The antidiarrheal activity of each crude extract was reported with the percentage of fecal output and diarrheal inhibition as described in the following formula [16, 17]:

(1) $$\begin{array}{c} \text{\% of inhibition }=\frac{\text{ average number of WFC}-\text{average number of WFT }}{\text{average number of WFC}}*100, \end{array}$$

where WFC = average number of wet feces in the control group and WFT = average number of wet feces in the test group, and

(2) $$\begin{array}{c} \text{percentage of fecal output}=\frac{\text{mean fecal weight of each group}}{\text{mean fecal weight of control}}*100. \end{array}$$

### 3.2. Preliminary Phytochemical Screening

The qualitative phytochemical investigations of the hydromethanol crude extracts of *Ruta chalepensis* and *Vernonia amygdalina* leaves were carried out using standard tests as described as follows. [18].

#### 3.2.1. Test for Terpenoids (Salkowski Test)

To 0.5 g of each of the crude extract leaves, 2 ml of chloroform was added. Then, 3 ml concentrated sulfuric acid was carefully added to form a layer. A reddish brown coloration of the interface indicates the presence of terpenoids.

#### 3.2.2. Test for Saponins

To 0.5 g of each of the crude extract leaves, 5 ml of distilled water was added in a test tube. Then, the solution was shaken vigorously and observed for a stable persistent froth. Formation of froth indicates the presence of saponins.

#### 3.2.3. Test for Tannins

About 0.5 g of each of the crude extract leaves was boiled in 10 ml of water in a test tube and then filtered. A few drops of 0.1% ferric chloride were added. A brownish green or a blue-black precipitate indicated the presence of tannins.

#### 3.2.4. Test for Flavonoids

About 10 ml of ethyl acetate was added to 0.2 g of each of the crude extract leaves and heated on a water bath for 3 min. The mixture was cooled and filtered. Then, about 4 ml of the filtrate was taken and shaken with 1 ml of dilute ammonia solution. The layers were allowed to separate, and the yellow color in the ammoniacal layer indicated the presence of flavonoids.

#### 3.2.5. Test for Cardiac Glycosides (Keller–Killiani Test)

To 0.5 g of each of the crude extract leaves diluted to 5 ml in water was added 2 ml of glacial acetic acid containing one drop of ferric chloride solution. This was underlayed with 1 ml of concentrated sulfuric acid. A brown ring at the interface indicated the presence of a deoxysugar characteristic of cardenolides. A violet ring may appear below the brown ring, while in the acetic acid layer a greenish ring may form just above the brown ring and gradually spread throughout this layer.

#### 3.2.6. Test for Steroids

Two ml of acetic anhydride was added to 0.5 g each of the crude extract leaves of each sample with 2 ml sulfuric acid. The color changed from violet to blue or green in some samples indicating the presence of steroids.

#### 3.2.7. Test for Alkaloids

0.5 g of the extract was diluted to 10 ml with acid alcohol, boiled, and filtered. To 5 ml of the filtrate, 2 ml of dilute ammonia and 5 ml of chloroform were added and shaken gently to extract the alkaloidal base. The chloroform layer was extracted with 10 ml of acetic acid. This was divided into two portions. Mayer's reagent was added to one portion and Dragendorff's reagent to the other. The formation of a cream (with Mayer's reagent) or a reddish brown precipitate (with Dragendorff's reagent) was regarded as positive for the presence of alkaloids.

### 3.3. Statistical Analysis

The data were entered and analyzed using the Statistical Package for the Social Sciences (SPSS) version 21.0 software. One-way analysis of variance (ANOVA) followed by Tukey's post hoc test was employed to analyze the difference between group means. The result was expressed as mean ± standard error of the mean (SEM), and *P* < 0.05 was considered as statistically significant.

### 3.4. Ethical Approval

Ethical clearance was obtained from the Ethical Review Board of Ambo University, College of Medicine and Health Sciences, Department of Pharmacy (protocol number: Phar/12/2017). After the experiment, the animals were ethically sacrificed with the use of sodium pentobarbital euthanasia.

## 4. Results

### 4.1. Effects on Castor Oil-Induced Diarrhea in Mice

In the castor oil-induced diarrheal model, the crude leaves extract of *Ruta chalepensis* leaves significantly prolonged the time of diarrheal onset and the frequency of stooling at 200 mg/kg.bw and 400 mg/kg.bw tested doses. Besides, the present study revealed that the percentage of diarrheal inhibition compared to controls was 23.6% (*P* < 0.05) and 51.31% (*P* < 0.05) at the doses of 200 and 400 mg/kg.bw, respectively. In addition, at the maximum tested doses (400 mg/kg.bw), the crude extract of *Ruta chalepensis* produced almost comparable effect (51.31%) with loperamide (62.8%). Nonetheless, at the dose of 100 mg/kg.bw, the extract did not have significant antidiarrheal effect in this model (Table 1).

The crude leaves extract of *Vernonia amygdalina* significantly prolonged the onset of diarrheal feces and decreased the frequency of wet feces defection only at the doses of 400 mg/kg.bw. This fraction produced the highest percentage of diarrheal inhibition (55.01%) at 400 mg/kg.bw, which was comparable with that of the standard drug (62.8 %). Contrastingly, the 100 mg/kg.bw and 200 mg/kg.bw tested doses of the extract did not possess significant antidiarrheal activity as compared to the negative control (Table 2).

As depicted in Table 1, there was a dose-dependent reduction in the percentage of mean fecal output among all tested doses of the crude extract *Ruta chalepensis* with 400 mg/kg.bw tested dose showing the highest effect (34%). Similarly, the crude leaves extract *Vernonia amygdalina* showed the maximum effect (31%) at the highest tested doses (400 mg/kg.bw) to lessen the percentage of mean fecal output (Table 2).

### 4.2. Preliminary Phytochemical Screening

Phytochemical screening test revealed that alkaloids, tannins, and saponins were detected in both crude extracts. However, flavonoids, cardiac glycosides, terpenoids, and steroids were exclusively found in crude leaves extracts of *Ruta chalepensis* (Table 3).

## 5. Discussion

Castor oil is the most commonly used diarrhea inducer in mice models. [19] Ricinoleic acid, the active metabolites of castor oil, is responsible for the diarrhea-inducing properties of castor oil. [20].

In the castor oil-induced diarrheal model, the crude leaves extract of *Ruta chalepensis* significantly prolonged the diarrheal onset and the frequency of stooling at middle and higher tested doses. This study is in line with other study which showed the hydromethanolic crude extract of *Indigofera spicata* at 200 and 400 mg/kg.bw doses had statistically significant inhibition of the frequency of defecation. [21] The significant antidiarrheal activity of this plant could be due to the probable localization of secondary metabolites to inhibit castor oil-induced fluid secretion. Besides, the crude leaves extract of *Ruta chalepensis* (200 and 400 mg/kg.bw) showed a significant percentage of diarrheal inhibition as compared to the controls which can further corroborate its antidiarrheal potential to undergo further investigation. Surprisingly, at the maximum tested doses (400 mg/kg.bw), the crude extract of *Ruta chalepensis* showed almost comparable effect with the standard drug. This dose-dependent effect of the crude extract may be probably due to the augmentation of the therapeutic concentration of the secondary metabolites as a result of increased dose. Nonetheless, at the dose of 100 mg/kg.bw, the extract was devoid of significant antidiarrheal effect in this model. Phytochemical screening study showed that the leaves extract of *Ruta chalepensis* was endowed with alkaloids, flavonoids, polyphenols, terpenoids, cardiac glycosides, and saponins. [22] Previous studies showed that terpenoids and steroids like phytosterols have a capacity to inhibit the production of prostaglandin E2 [23, 24], which has a crucial role in the stimulation of intestinal secretions. [25, 26] Accordingly, the significant antidiarrheal activity observed in the *Ruta chalepensis* crude extract at different doses could probably be linked to the presence of these secondary metabolites in the crude extract. Besides, Moazedi et al. [27] reported that the hydroalcoholic leaves extract of *Ruta chalepensis* had a significant antispasmodic effect on rat ileum. This could probably suggest its antidiarrheal effect by inhibition of castor oil-induced gastrointestinal motility.

The crude leaves extract of *Vernonia amygdalina* had shown significant antidiarrheal activity by extending the onset of diarrhea and reducing the frequency of defection only at a maximum tested dose (400 mg/kg.bw). This could probably suggest the low potency of this medicinal plant as compared to the *Ruta chalepensis* leaves extract. Contrastingly, the lower and middles doses of the extract did not have significant antidiarrheal activity on castor oil-induced diarrhea as compared to the negative control, which could be probably due to low concentration of bioactive metabolites at lower and middle tested doses.

The present study showed that maximum reduction in the percentage of the mean fecal output was achieved at the maximum tested dose (400 mg/kg.bw) in both extracts. This could suggest the probable localization of the active ingredients in the uppermost doses of the crude extracts of both medicinal plants.

Although the phytochemical constituents responsible for the antidiarrheal effect are yet to be identified, and the amount of phytochemical constituents that are responsible for impeding gastrointestinal motility such as tannins [28, 29] and alkaloids [7] appear to increase with dose. This could possibly be the reason why significant antidiarrheal effect was observed at the higher doses of the crude extracts of both medicinal plants. However, at the lower and middle doses of the crude leaves extract of *Vernonia amygdalina*, the significant antidiarrheal effect was not observed in this model. This might be due to the lack of secondary metabolites such as terpenoids [24], steroids [23], and flavonoids [30, 31] that could inhibit castor oil-induced fluid secretion in the intestine.

## 6. Conclusion

The present study demonstrated that the crude leaves extract of *Ruta chalepensis* (200 and 400 mg/kg.bw) and *Vernonia amygdalina* (400 mg/kg.bw) possessed significant antidiarrheal activity due to its inhibitory effect on castor oil-induced gastrointestinal fluid secretion. The antidiarrheal activities of these crude extracts could probably be attributed to the presence of bioactive agents in both medicinal plants.

## Figures and Tables

**Table 1:** Effect of the crude extract of *Ruta chalepensis* leaves on castor oil-induced diarrheal model in mice.

Group	Onset time of diarrhea (min)	Total # of wet feces in 4 h	Total weight of feces (gm)	% Inhibition of defecation	% Mean fecal output
Control	87.33 ± 29.96	2.67 ± 0.67	1.85 ± 0.24	—	—
Loperamide 3 mg/kg	234.3 ± 3.81^*∗*^	1 ± 0.52^*∗*^	0.55 ± 0.18^*∗*^	62.8	30
CE100 mg/kg	82.8 ± 25	2.3 ± 0.62	1.03 ± 0.25	13.86	56
CE200 mg/kg	93.5 ± 40^*∗*^	3.3 ± 0.84^*∗*^	0.98 ± 0.24^*∗*^	23.6	53
CE400 mg/kg	206.3 ± 31.53^*∗*^	1.3 ± 0.67^*∗*^	0.63 ± 0.11^*∗*^	51.31	34

*Note.* Values are expressed as mean ± SEM (*n* = 6), and analysis was performed with one-way ANOVA followed by the Tukey test, ^*∗*^*P* < 0.05. CE = crude extract, and controls received 10 ml/kg of 2% Tween 80 in distilled water. SEM: standard error of the mean and ANOVA: analysis of variance.

**Table 2:** Effect of the crude extract of *Vernonia amygdalina* leaves on castor oil-induced diarrheal model in mice.

Group	Onset time of diarrhea (min)	Total # of wet feces in 4 h	Total weight of feces (gm)	% Inhibition of defecation	% Mean fecal output
Control	87.33 ± 29.96	2.67 ± 0.67	1.85 ± 0.24	—	—
Loperamide 3 mg/kg	234.3 ± 3.81^*∗*^	1 ± 0.52^*∗*^	0.55 ± 0.18^*∗*^	62.8	30
100 mg/kg	40.8 ± 28.3	2.5 ± 0.8	1.2 ± 0.25	6.4	65
200 mg/kg	128 ± 44.7	2.3 ± 0.49	0.9 ± 0.31	13.85	49
400 mg/kg	156.8 ± 38^*∗*^	1.2 ± 0.8^*∗*^	0.58 ± 0.12^*∗*^	55.01	31

*Note.* Values are expressed as mean ± SEM (*n* = 6), and analysis was performed with one-way ANOVA followed by the Tukey test, ^*∗*^*P* < 0.05; CE = crude extract, and controls received 10 ml/kg of 2% Tween 80 in distilled water. SEM: standard error of the mean and ANOVA: analysis of variance.

**Table 3:** Preliminary phytochemical screening of hydromethanol crude extracts of *Ruta chalepensis* and *Vernonia amygdalina* leaves.

Secondary metabolites	*Ruta chalepensis* crude extract	*Vernonia amygdalina* crude extract
Alkaloids	+	+
Tannins	+	+
Saponins	+	+
Terpenoids	+	−
Steroids	+	−
Flavonoids	+	−
Anthraquinones	−	−
Cardiac glycosides	+	−

+ = present and − = absent.

## Data Availability

The datasets used and/or analyzed during the current study will be obtained from the corresponding and the first author of this project.
